# Correction: Replication Study: BET bromodomain inhibition as a therapeutic strategy to target c-Myc

**DOI:** 10.7554/eLife.34572

**Published:** 2018-01-08

**Authors:** Fraser Aird, Irawati Kandela, Christine Mantis, Reproducibility Project: Cancer Biology

Aird F, Kandela I, Mantis C, Reproducibility Project: Cancer Biology. 2017. Replication Study: BET bromodomain inhibition as a therapeutic strategy to target c-Myc. *eLife* **6**:e21253. doi: 10.7554/eLife.21253.

Published 19, January 2017

This is a corrigendum for Replication Study: BET bromodomain inhibition as a therapeutic strategy to target c-Myc.

The calculation of variance in the meta-analysis of the overall survival distributions for mice treated with (+)-JQ1 vs. mice treated with vehicle control (Figure 4B) is incorrect. Subsequently, the effect size and the associated *p* value of the meta-analysis are incorrect. The effect size (Hazard Ratio) for this comparison should be HR = 9.16 95% CI [1.39, 60.54] (instead of the reported HR = 13.12 95% CI [1.79, 96.02]) resulting in a p value of *p* = 0.0215 (instead of the reported *p* = 0.0112).

The language surrounding this comparison in the Results and Discussion section as well as in Figure 4 and the Figure Legend (Figure 4B) have been amended to account for this correction. Additional r code for the meta-analysis can be found on the Open Science Framework: https://osf.io/x6g87/.

The corrected Figure 4 is shown here:

**Figure 1:**
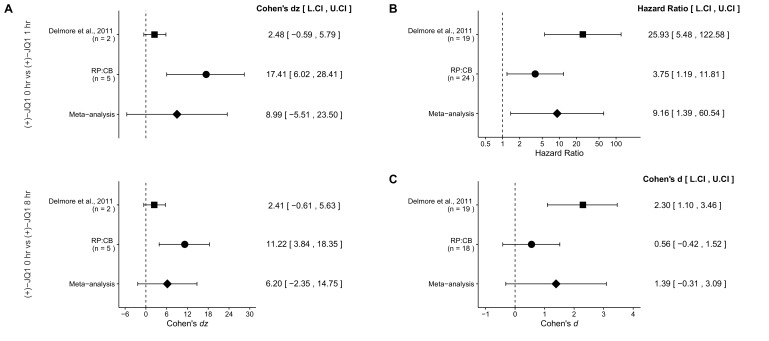


The originally published Figure 4 is also shown for reference:

**Figure 2:**
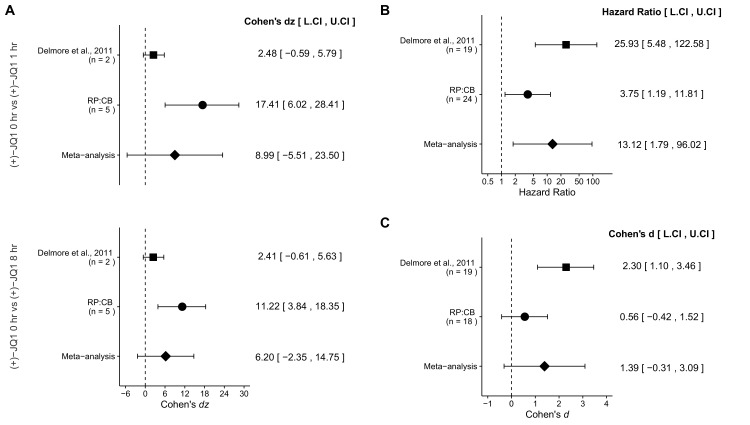


The article has now been corrected.

